# Sucking and drinking behaviour in preweaned dairy calves in the first five weeks of life

**DOI:** 10.1186/s12917-022-03280-x

**Published:** 2022-05-13

**Authors:** Ueli Braun, Manon Kochan, Martin Kaske, Christian Gerspach, Ulrich Bleul

**Affiliations:** Department of Farm Animals, Vetsuisse Faculty, University of Zurich, Winterthurerstrasse 260, CH-8057 Zurich, Switzerland

**Keywords:** Calf, Feeding, Milk, Swallowing, Sucking, Drinking duration, Drinking speed

## Abstract

**Background:**

Nursing and sucking are essential for adequate nourishment of preweaned calves and the relationship between sucking indices has not been studied. The goal of this study was to investigate the number of sucks per litre of milk and per minute of drinking and the amount of milk ingested per suck in healthy preweaned calves. Correlation coefficients were calculated for the relationships between these variables. Eighteen healthy calves were used from birth to 5 weeks of age, and five measurements were made at the end of weeks 1 to 5. The calves were randomly divided into three groups and offered milk twice daily in a bucket with a rubber nipple. The amount of milk offered per day was equal to 12% of body weight in group A and 16% of body weight in group B. Calves in group C were offered as much milk as they wanted during each feeding period. The duration of drinking was determined with a stopwatch, and the number of sucks was counted with a handheld tally counter. The variables drinking duration, total amount consumed and the number of sucks required were used to calculate the number of sucks/min, the number of sucks/L, the amount ingested per suck and drinking speed.

**Results:**

The number of sucks/min ranged from 113 to 133 and increased significantly during the study period. The mean number of sucks/L decreased from 204 in week 1 to 141 in week 5 and drinking speed increased from 0.6 to 1.0 L/min. There were significant correlations between the number of sucks/L of milk and the amount of milk ingested per suck, drinking duration, total amount consumed and drinking speed. Drinking speed was positively correlated with the amount of milk ingested per suck and the total amount of milk consumed, and negatively correlated with drinking duration.

**Conclusions:**

These findings show that drinking variables of calves offered different amounts of milk vary little and significant changes occur during the same period with respect to the number of sucks/L of milk and the amount of milk ingested per suck. Several drinking variables are significantly correlated with other variables.

**Supplementary Information:**

The online version contains supplementary material available at 10.1186/s12917-022-03280-x.

## Background

Nursing and sucking are essential for adequate nourishment of preweaned calves. Calves may ingest milk from the dam’s udder or milk or milk replacer from a bucket [1], a bucket with a rubber nipple [2], a teat bottle [3], a milk bar or an automated feeder [4–6]. A recent review investigated calf management and nutrition for the past 100 years [7]. When calves drinking milk from a bucket with a nipple, the position of the nipple and the size of the nipple opening are crucial. Nursing from a nipple attached near the bottom of the bucket requires less sucking effort than nursing from a nipple attached near the top and connected to the bottom of the bucket through a rubber hose. Likewise, a small nipple opening requires a stronger sucking effort than a large opening. Oesophageal groove reflex dysfunction is a potential problem associated with the consumption of milk and can result in milk entering the reticulorumen instead of the abomasum [8]. This complication is referred to as ruminal drinker syndrome and causes rumen acidosis accompanied by D-lactic acidosis; clinical signs vary in severity but primarily include depression and ataxia [9]. Force-feeding milk with a stomach tube [10] and possibly reflux of milk from the abomasum into the reticulorumen [8] can also cause ruminal drinker syndrome. Spontaneously ingested meal sizes representing up to 13.2% of body weight did not result in backflow of milk into the reticulorumen in calves [3], and the oesophageal groove reflex was not adversely affected by feeding cold milk [2, 3] or by various factors related to feeding management including nipple position on the bucket or size of the nipple opening [2]. The feeding process can be quantified by measuring the amount of milk consumed, the duration of drinking and the drinking speed; several authors have investigated these variables in calves using automated feeders [4, 6]. With respect to the amount of milk fed per day, feeding practices have evolved greatly over the decades. Recommendations range from 8 to 12, 14, 16 and 20% of body weight to ad libitum intake [11–13], or 4 to 6 L of milk or milk replacer given in two meals in an open bucket or a bucket with a rubber nipple [1]. Amounts representing 8 or 10% of body weight do not meet the nutritional requirements of preweaned calves [13]. Large-volume milk feeding was thought to be associated with health problems [11], but studies of calves fed ad libitum have shown that this is not accurate [11, 14]. Likewise, complications such as reflux of milk into the reticulorumen or diarrhoea did not occur in calves that were fed milk at 13.2% of body weight daily [13]. Large-volume milk feedings improve weight gain and the immediate general condition and health of the calf. In addition, positive effects on the future production and fertility performance of dairy cows via metabolic programming may be realised [15]. Limiting milk intake leads to hungry calves and adversely affects their growth, health and wellbeing. It also favours ethopathies such as cross-sucking of the ears, penis, navel or scrotum among calves [1].

The feeding behaviour of calves is affected by various diseases, but changes in behaviour vary with the type of illness [4]. Drinking speed, daily consumption and number of unrewarded visits to the feeder were reduced in calves with diarrhoea, whereas these variables were less severely affected by respiratory disease. The negative effect on these feeding variables was noticed as early as 4 days before calves had diarrhoea and remained apparent for seven to 10 days after treatment [4]. Similar observations were made with respect to daily milk consumption in calves experimentally infected with rotavirus [5]. Drinking speed was the most sensitive feeding variable for the early detection of sick calves but there was no clear advantage to monitoring this variable compared with direct daily monitoring of the calves by barn staff [16]. Calves with subclinical respiratory disease had similar drinking speeds, daily milk consumption and number of visits to the feeder as healthy calves, but calves with clinical respiratory disease had lower drinking speeds than healthy calves [6]; however, the sensitivity and specificity of these feeding variables were not sufficient for early detection of respiratory diseases [17]. Drinking speed did not differ between healthy calves and those with mild disease [18]. Data collected from automated feeders were not suitable as the sole detection method for neonatal calf diarrhoea and bovine respiratory disease but they may serve as a useful preliminary tool for disease detection in calves that require further monitoring by farm personnel [19].

To the authors‘knowledge, the relationship between sucking indices, which include number of sucks/min, number of sucks/L and amount of milk ingested per suck, and the variables daily consumption, drinking duration per meal and drinking speed has not been studied in preweaned calves. Detailed information about sucking indices is not available and therefore our goal was to investigate the amount of milk ingested per suck (or number of sucks required to ingest a given amount of milk) and the number of sucks/min in calves fed whole milk from a nipple attached to the bottom of a bucket. A secondary goal was to examine whether these measures vary within and between groups of calves that are fed different amounts of milk per day, and whether correlations exist between different drinking variables.

## Methods

### Animals

Eighteen healthy Holstein Friesian male calves were used. Measurements were made on two consecutive days during the morning feeding period when the calves were 1, 2, 3, 4 and 5 weeks of age; this corresponded to days 7/8, 14/15, 21/22, 28/29 and 35/36. The birth weight was 44.8 ± 6.83 kg (mean ± sd) and did not differ among the groups. The calves were born in the summer (*n* = 11), autumn (*n* = 3) and winter (*n* = 4). The calves were assigned to three groups of six calves each (A, B, C), and each group was offered a different amount of milk. The calves were kept in individual pens but could see other calves, and water and hay were available at all times. The calves were not given concentrated feed until the end of the experiment. Details are described in a dissertation [20].

### Feeding and groups

The newborn calves received two bottle feedings of 2 to 3 l (depending on appetite) of colostrum. Thereafter they were fed twice a day at 0700 and 1600 with cow’s milk heated to 39.0 °C and offered in a bucket with a rubber nipple attached to the bottom of the bucket (Hauptner, Langenthal). The nipple had a medium-sized cross-like opening. The calves were weighed on days 7, 14, 21, 28 and 35 days of age, early in the morning before feeding to calculate the amount of milk offered daily in the following week. The daily amount of milk fed was 12% of body weight in group A and 16% of body weight in group B, divided into two equal meals. Calves of group C were allowed to consume as much milk as they wanted at the two feedings. In all three groups the buckets were removed when the calves stopped drinking for > 30 seconds. Residual milk in the bucket was used to calculate the amount consumed, which was expressed as percentage of the body weight. The duration of drinking was determined with a stopwatch, each clicking noise from the check valve of the nipple was interpreted as one suck (video 1), and the number of sucks was counted with a handheld tally counter. The duration of drinking, the amount consumed and the total number of sucks were used to calculate the number of sucks/min (total number of sucks per duration of drinking), the number of sucks/L of milk (total number of sucks per total consumed amount), the amount of milk ingested per suck (total amount consumed per total number of sucks) and the drinking speed (amount consumed per duration of drinking).

### Statistical analysis

The program SPSS Version 27 (IBM SPSS Statistics 27.0, Switzerland) was used for analysis. The means were calculated for the measurements from the consecutive days and the values referred to as weeks 1, 2, 3, 4 and 5. The Shapiro-Wilk test was used to test for normality. Normal data are represented as mean ± standard deviation and non-normal values as median and range and as boxplots. The general linear model, choosing ANOVA with repeated measures and replacing *polynomial* with *difference,* was used to analyse changes in the variables over time. ANOVA and Bonferroni post-hoc test were used for pair-wise comparison of variables from different weeks. Pearson’s correlation coefficients were calculated to describe the correlation between different variables, which were presented as scatter plots. Differences were considered significant at *P* < 0.05.

## Results

### Body weight

The mean body weight of all calves increased from 49.7 kg in week 1 to 74.2 kg in week 5 (*P* < 0.01) (Table 1). There were significant correlations between the body weights at different time points (Table 2).

**Table 1:** Body weight and drinking variables in 18 Holstein Friesian calves aged 1 to 5 weeks (means±standard deviations)

		Age (weeks)				
Variable	Group	1	2	3	4	5
Body weight	A	50.0 ± 3.2	55.0 ± 3.7	59.3 ± 5.5	67.3 ± 3.9	72.5 ± 3.8
(kg)	B	49.8 ± 5.5	54.7 ± 6.6	60.5 ± 5.9	67.3 ± 7.2	73.9 ± 8.2
	C	49.2 ± 10.7	54.7 ± 10.9	60.2 ± 14.5	66.5 ± 14.8	76.3 ± 17.3
	All calves**	49.7 ± 6.76	54.8 ± 7.19	60.0 ± 9.02	67.1 ± 9.14	74.2 ± 10.7
Amount of	A	3.0 ± 0.23^2^	3.3 ± 0.20^1,2^	3.5 ± 0.32	4.1 ± 0.19	4.3 ± 2.65
milk	B	3.7 ± 0.79	4.4 ± 0.52	4.7 ± 0.55	5.1 ± 0.72	4.6 ± 1.13
consumed	C	4.0 ± 0.44	4.3 ± 0.82	4.6 ± 1.45	5.5 ± 2.01	5.5 ± 1.32
per meal (L)	All calves**	3.6 ± 0.67	4.0 ± 0.72	4.2 ± 1.01	4.9 ± 1.33	4.8 ± 1.08
Amount of	A	6.0 ± 0.07^2^	6.0 ± 0.06^1,2^	5.9 ± 0.05^1,2^	6.0 ± 0.04^1,2^	6.0 ± 0.07
milk	B	7.4 ± 0.97	7.9 ± 0.07	7.7 ± 0.64	7.6 ± 6.71	6.3 ± 17.19
consumed per	C	8.3 ± 12.12	7.9 ± 13.82	7.5 ± 10.63	8.2 ± 15.01	7.2 ± 9.18
meal in percent of body weight	All calves	7.3 ± 12.92	7.3 ± 11.87	7.1 ± 10.67	7.3 ± 12.97	6.5 ± 11.79
Drinking	A	4.9 ± 1.09	3.9 ± 1.09^2^	4.5 ± 1.49	4.7 ± 1.85	4.4 ± 1.05
duration per	B	6.0 ± 2.02	5.0 ± 1.31	4.8 ± 1.13	5.2 ± 1.07	5.5 ± 0.63
meal (min)	C	7.5 ± 1.61	6.4 ± 1.17	5.7 ± 0.92	6.1 ± 2.01	5.8 ± 1.09
	All calves	6.4 ± 1.76	5.1 ± 1.54	5.0 ± 1.25	5.4 ± 1.70	5.2 ± 1.08
Drinking	A	0.6 ± 0.10	0.9 ± 0.07	0.9 ± 0.36	1.0 ± 0.37	1.1 ± 0.27
speed	B	0.7 ± 0.24	0.8 ± 0.31	1.0 ± 0.30	1.1 ± 0.40	0.8 ± 0.21
(L/min)	C	0.6 ± 1.51	0.9 ± 0.32	0.8 ± 0.26	1.0 ± 0.43	1.0 ± 0.35
	All calves**	0.6 ± 0.18	0.9 ± 0.28	0.9 ± 0.31	1.0 ± 0.38	1.0 ± 0.28
Sucks/min	A	126 ± 14	135 ± 10	144 ± 17^2^	143 ± 10	141 ± 9
	B	111 ± 13	124 ± 26	133 ± 21	136 ± 16	115 ± 29
	C	103 ± 31	100 ± 30	110 ± 14	120 ± 18	122 ± 17
	All calves*	113 ± 22	120 ± 27	129 ± 22	133 ± 17	126 ± 22
Sucks/L	A	230 ± 22	157 ± 38	183 ± 66	167 ± 57	143 ± 36
of milk	B	189 ± 86	138 ± 42	139 ± 36	141 ± 36	142 ± 30
	C	198 ± 86	152 ± 70	147 ± 38	147 ± 62	137 ± 44
	All calves**	204 ± 71	149 ± 50	156 ± 50	151 ± 51	141 ± 35
Amount of	A	4.6 ± 0.55	6.8 ± 1.60	6.1 ± 2.13	6.9 ± 2.52	7.5 ± 1.70
milk ingested	B	5.9 ± 1.70	8.1 ± 2.48	7.7 ± 1.63	7.6 ± 2.26	7.4 ± 1.49
per suck (ml)	C	6.2 ± 2.58	7.6 ± 2.88	7.5 ± 1.78	8.3 ± 3.65	8.0 ± 2.96
	All calves*	5.6 ± 1.89	7.5 ± 2.31	7.1 ± 1.90	7.6 ± 2.77	7.6 ± 2.04

^1^ Difference A:B *P* < 0.05

^2^ Difference A:C *P* < 0.05

* Significant changes during study period *P* < 0.05

** Significant changes during study period *P* < 0.01

**Table 2:** Significant correlations between measurements at different measuring times for all groups combined (* *P* < 0.05, ** *P* < 0.01)

	Correlations coefficients			
Variable	Between week 1 and …	Between week 2 and …	Between week 3 and …	Between week 4 and …
Body weight	W2: 0.96** W3: 0.91** W4: 0.92** W5: 0.89**	W3: 0.94** W4: 0.97** W5: 0.95**	W4: 0.96** W5: 0.94**	W5: 0.96**
Amount of milk consumed/meal	W2: 0.76** W3: 0.64** W4: 0.65**	W3: 0.68** W4: 0.76** W5: 0.58*	W4: 0.88** W5: 0.58*	W5: 0.76**
Duration of drinking/meal	W2: 0.62**	3: 0.55* 4: 0.49*	4: 0.75** 5: 0.67**	W5: 0.67**
Drinking speed	W2: 0.66** W4: 0.51*	W3: 0.50* W4: 0.51*	W4: 0.82** W5: 0.61**	W5: 0.71**
Sucks/min	W2: 0.60** W4: 0.54**	W3: 0.48* W4: 0.50* W5: 0.53**	W4: 0.76* W5: 0.61**	W5: 0.66**
Sucks/L of milk	W2: 0.70** W3: 0.53* W4: 0.48* W5: 0.48*	W3: 0.61*	W4: 0.61** W5: 0.70**	W5: 0.73**
Amount of milk ingested/suck	W2: 0.73**	W3: 0.65**	W4: 0.75**	W5: 0.77**

W Week

### Milk consumption per meal

**Fig. 1:**
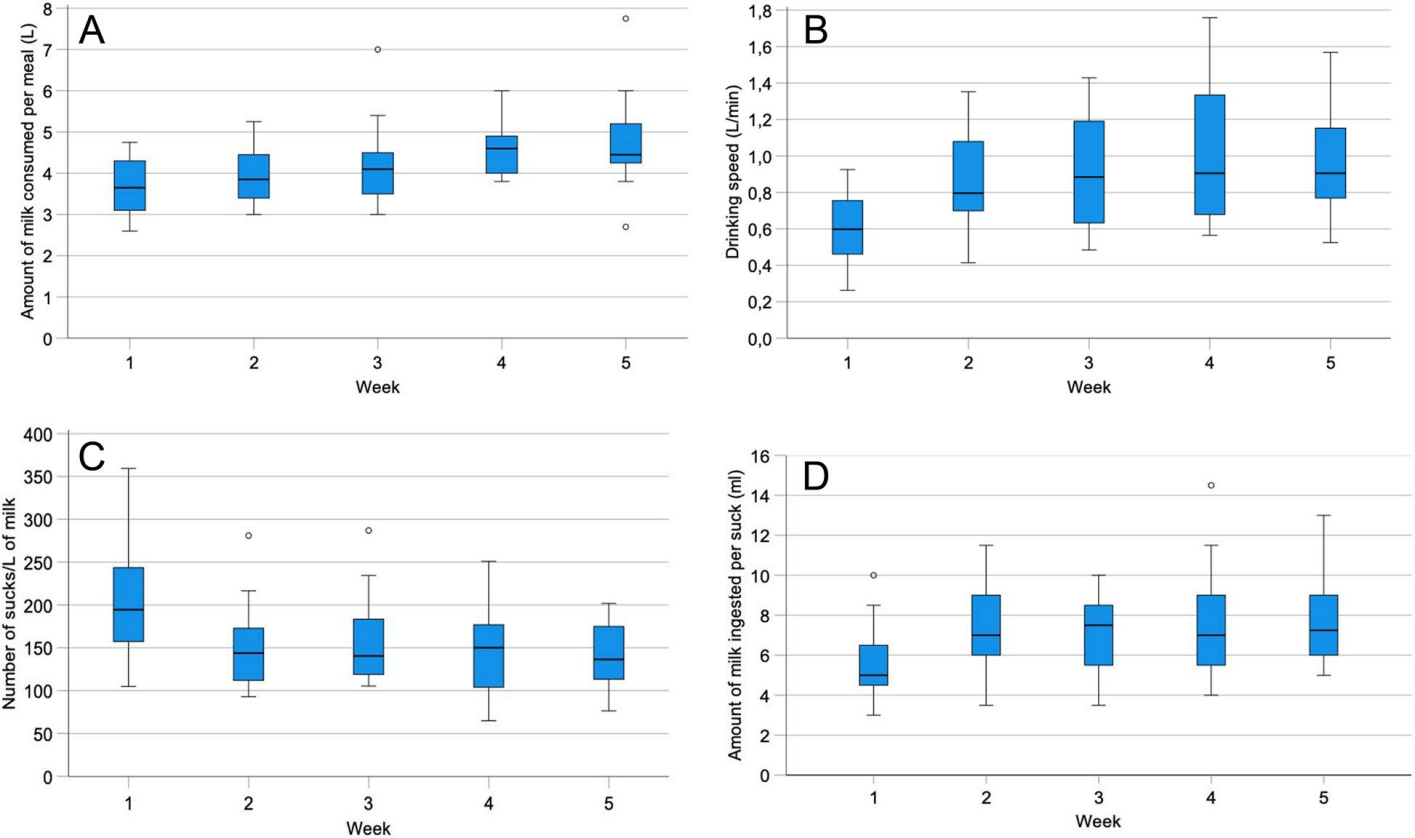
Amount of milk consumed per meal (**A**), drinking speed (**B**), number of sucks/ L of milk (**C**) and amount of milk ingested per suck (**D**) in 18 Holstein Friesian calves from 1 to 5 weeks of age. Boxplot presentation as described by Field [21]: Within the box, the thick horizontal line shows the median. The top and bottom of the blue box represent the upper and lower quartiles, respectively. The distance between the top of the box and the top of the whisker shows the range of the top 25% of scores. Similarly, the distance between the bottom of the box and the end of the bottom whisker shows the range of the lowest 25% of scores. ° = Outliers

The mean amount of milk consumed per meal increased from 3.6 L in week 1 to 4.8 L in week 5 (*P* < 0.01) (Table 1, Fig. 1A), representing 5.9 to 6.0% of body weight in group A, 6.3 to 7.9% in group B and 7.2 to 8.3% in group C. The amounts of milk relative to body weight differed significantly among the groups in the first 4 weeks (*P* < 0.05). There were significant correlations between the amounts of milk consumed at different time points (Table 2).

### Drinking duration per meal

The mean duration ranged from 5.0 (week 3) to 6.4 minutes (week 1) (Table 1) and did not change significantly during the study period. A significant difference between groups occurred in week 2 (groups A and C, *P* < 0.05). There were significant correlations between the drinking durations at different time points (Table 2).

### Drinking speed

The mean drinking speed increased significantly from 0.6 L/min in week 1 to 1.0 L/min in week 5 (*P* < 0.01) (Table 1, Fig. 1B) but did not differ among groups. There were significant correlations between the drinking speeds at different time points (Table 2).

### Number of sucks/min

The mean number of sucks/min varied from 113 (week 1) to 133 (week 4) and increased (*P* < 0.05) during the study period (Table 1). The number of sucks/min differed (*P* < 0.05) between groups A (144) and C (110) in week 3, and in the remaining weeks, it tended to be larger in group A than in groups B and C. There were significant correlations between the numbers of sucks at different time points (Table 2).

### Number of sucks/L of milk

The number of sucks/L of milk decreased significantly from 204 in week 1 to 141 in week 5 (*P* < 0.01) (Table 1, Fig. 1C), and did not differ among groups. There were significant correlations between the numbers of sucks/L of milk at different time points (Table 2).

### Amount of milk ingested per suck

The amount of milk ingested per suck increased from 5.6 ml in week 1 to 7.6 ml in week 5 (*P* < 0.05) (Table 1, Fig. 1D). The groups did not differ significantly. There were significant correlations between the amounts of milk ingested per suck at different time points (Table 2).

### Correlations between the number of sucks/min and other drinking variables

**Fig. 2:**
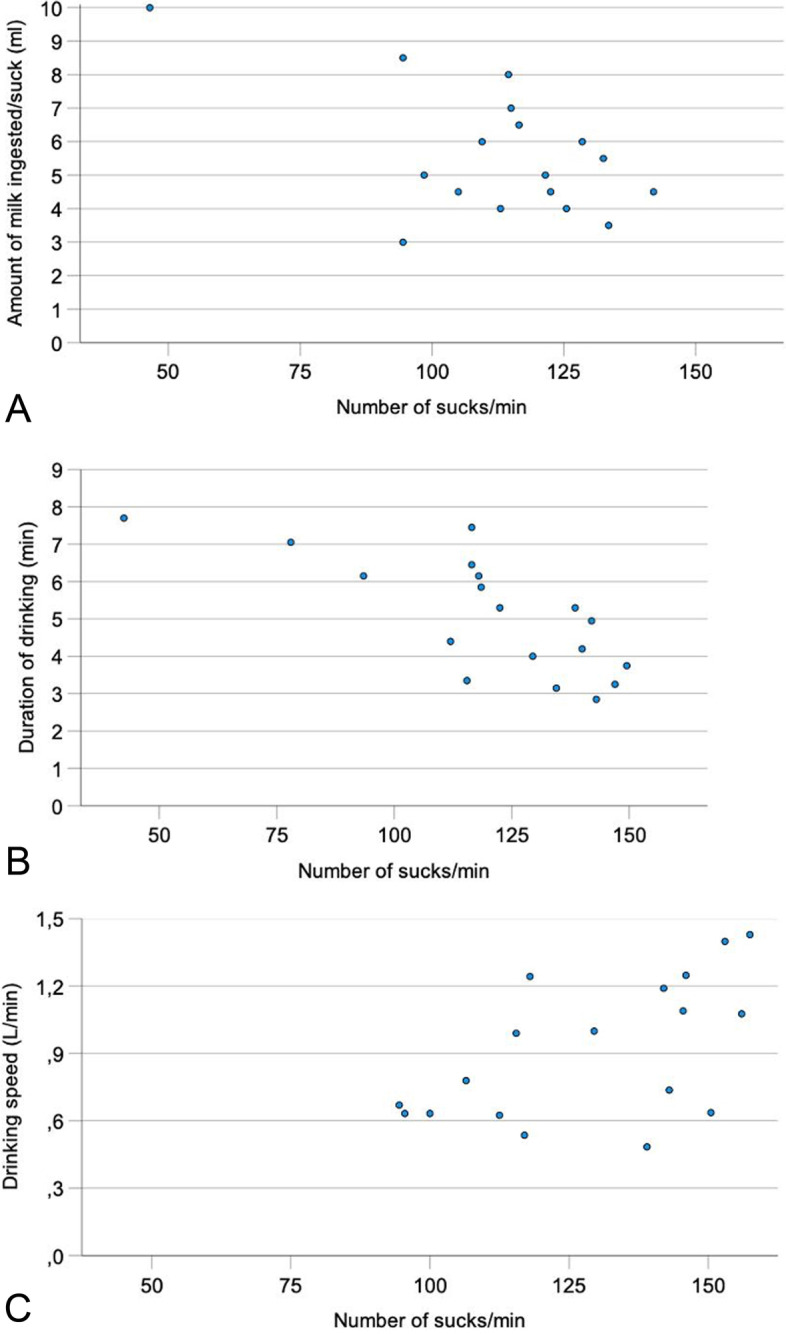
Relationship between the number of sucks/min and amount of milk ingested per suck in week 1 (**A**), duration of drinking in week 2 (**B**) and drinking speed in week 3 (**C**) in 18 Holstein Friesian calves

The number of sucks/min was negatively correlated with the amount of milk ingested per suck in week 1 (*r* = − 0.57*) (Table 3, Fig. 2A, week 1) and with drinking duration in weeks 2 and 3 (*r* = − 0.73** and *r* = − 0.68**) (Fig. 2B, week 2). There were significant correlations between the number of sucks/ min and drinking speed in weeks 2 and 3 (*r* = 0.49* and *r* = 0.56*) (Fig. 2C, week 3).

**Table 3:** Significant correlations between various drinking variables for all groups combined (* *P* < 0.05, ** *P* < 0.01)

	Correlation coefficients					
Variable	Sucks/min	Sucks/L of milk	Amount of milk/suck	Drinking duration/meal	Amount consumed/ meal	Drinking speed
Sucks/min	–	NSC	W1: −0.57*	W2: −0.73** W3: −0.68**	NSC	W3: 0.56*
Sucks/L of milk		–	W1: −0.91** W2: −0.94** W3: −0.96** W4: − 0.94** W5: − 0.95**	W1: 0.63** W3: 0.51* W4: 0.76** W5: 0.61*	W1: − 0.52* W2: − 0.51* W3: − 0.62** W4: − 0.49* W5: − 0.51*	W1: − 0.77** W2: − 0.63** W3: − 0.79** W4: − 0.89** W5: − 0.80**
Amount of milk/suck			–	W4: −0.61** W5: − 0.53*	W3: 0.69** W4: 0.69** W5: 0.60**	W1: 0.59* W2: 0.56* W3: 0.81** W4: 0.93** W5: 0.82**
Drinking duration/meal				–	NSC	W1: −0.78** W2: − 0.82** W3: − 0.78** W4: − 0.72** W5: − 0.67**
Amount consumed/meal					–	W1: 0.60* W3: 0.54* W4: 0.61** W5: 0.66**
Body weight	NSC	W3: −0.54* W4: − 0.57* W5: − 0.57*	W3: 0.63** W4: 0.69** W5: 0.66**	NSC	W1: 0.57* W2: 0.55* W3: 0.79** W4: 0.73** W5: 0.61**	W3: 0.58* W4: 0.73** W5: 0.54*

NSC No significant correlations

W Week

### Correlations between the number of sucks per litre of milk and other drinking variables

**Fig. 3:**
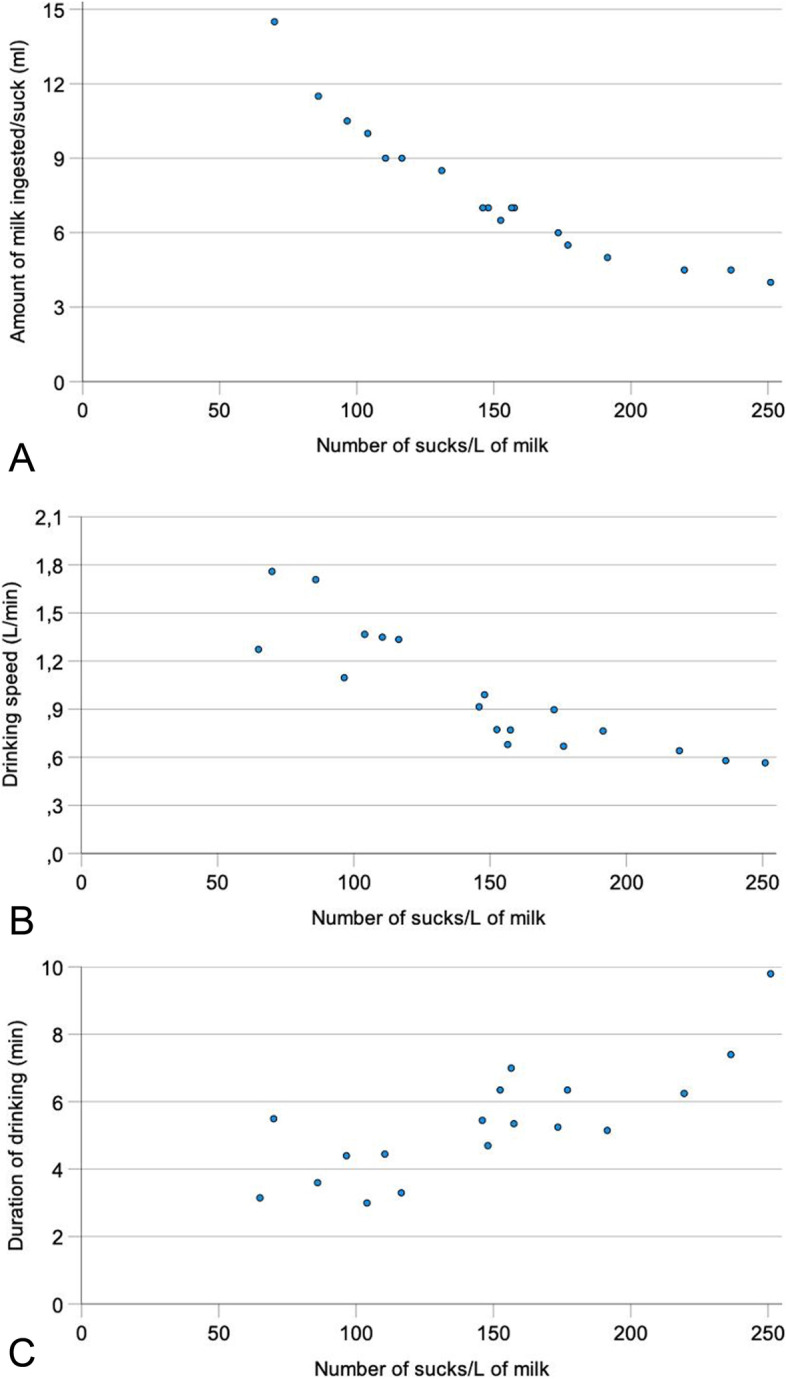
Relationship between the number of sucks/L of milk and the amount of milk ingested per suck (**A**), drinking speed (**B**) and drinking duration in week 4 (**C**) in 18 Holstein Friesian calves

At all measuring times, the number of sucks/L of milk was negatively correlated with the amount of milk ingested per suck (*r* = − 0.91** to *r* = − 0.96**) (Table 3; Fig. 3A, week 4), the amount of milk consumed per meal (*r* = − 0.51* to *r* = 0.62**) and drinking speed (Fig. 3B, week 4) (*r* = − 0.63* to *r* = − 0.89**). The number of sucks/L of milk was positively correlated with the duration of drinking (Fig. 3C, week 4) (*r* = 0.51* to *r* = 0.76**).

### Correlations between the amount of milk ingested per suck and other drinking variables

**Fig. 4:**
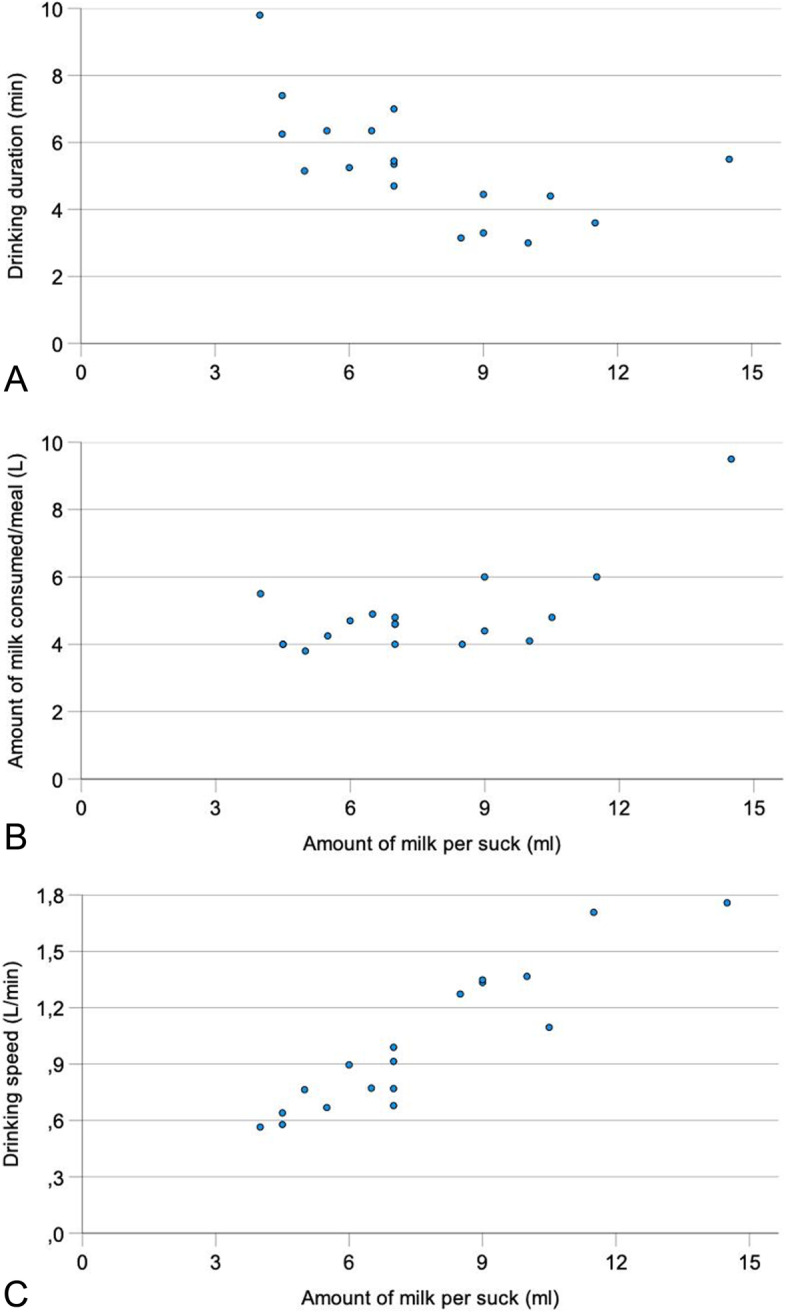
Relationship between the amount of milk ingested per suck and drinking duration (**A**), amount of milk consumed per meal (**B**) and drinking speed (**C**) in week 4 in 18 Holstein Friesian calves. Figure 4 only shows 16 dots instead of 18 because two calves each had identical values, which are superimposed (calves 2 and 3, amount consumed per meal 4.0 L, amount ingested per suck 4.5 ml; calves 7 and 15, amount consumed per meal 4.6 L, amount ingested per suck 7.0 ml

In addition to correlations described above (Figs. 2A and 3A), the amount of milk ingested per suck was negatively correlated with the duration of drinking (*r* = − 0.53** and *r* = − 0.61**) (Fig. 4A, week 4) and positively correlated with the amount of milk ingested per meal (*r* = 0.60** to 0.69**) (Fig. 4B, week 4) and the drinking speed (*r* = 0.56* to *r* = 0.93**) (Table 3; Fig. 4C, week 4).

### Correlations between drinking duration per meal and other drinking variables

In addition to correlations described above (Figs. 2B, 3C and Fig. 4A; Table 3), drinking duration per meal was negatively correlated with the drinking speed (*r* = − 0.67** to *r* = − 0.82**) at all measuring times.

### Correlations between amount of milk consumed per meal and other drinking variables

In addition to correlations described above (Fig. 4B, Table 3), the amount of milk consumed per meal was significantly correlated with the drinking speed (*r* = 0.54* to *r* = 0.66**) at four measuring times.

### Correlations between drinking speed and other drinking variables

There were significant correlations between the drinking speed and sucks/min (*r* = 0.56*) (Table 3, Fig. 2C, week 3), sucks/L of milk (*r* = − 0.63** to *r* = − 0.89**) (Fig. 3B, week 4), amount of milk ingested per suck (*r* = 0.56* to *r* = 0.93**) (Fig. 4C, week 4), drinking duration per meal (*r* = − 0.67** to *r* = − 0.82**) and amount of milk consumed per meal (*r* = 0.54* to *r* = 0.66**).

### Correlations between body weight and drinking variables

Body weight was significantly correlated with the number of sucks/L of milk (*r* = − 0.54* to *r* = − 0.57*), amount of milk ingested per suck (*r* = 0.63** to *r* = 0.69**), amount of milk consumed per meal (*r* = 0.55* to *r* = 0.79**) and drinking speed (*r* = 0.54* to *r* = 0.73**) at several measuring times (Table 3).

## Discussion

The amount of milk consumed per day, the duration of drinking and the drinking speed in artificially reared calves have been described by several authors [4, 6, 16, 17]. To our knowledge, the present study is the first to analyse the number of sucks/min, the number of sucks/L of milk and the amount of milk ingested per suck in calves fed whole milk. However, only the quantification of sucks represents a novel variable because the *number of sucks per litre* and *per minute* can be derived from the number of sucks, the amount of milk consumed and the drinking duration. It is important to point out that sucking is not necessarily linked to simultaneous swallowing. In contrast to calves, the relationship between sucking and swallowing and the suck:swallow ratio have been investigated in human babies [22, 23]. The suck:swallow ratio of bottle-fed babies varies from 1:1 to 4:1 because babies are able to accumulate milk from more than one suck in their mouth before swallowing the liquid in one swallowing movement [22]. A suck:swallow ratio of 1:1 occurred significantly more often when a high-flow bottle teat was used, and a ratio of 4:1 was significantly more common with a low-flow bottle teat [22]. Flexible endoscopy and video-fluoroscopy are the most common techniques for the assessment of swallowing [23]; the latter is considered the gold standard because it allows visualisation of all phases of swallowing as well as the airways. To our knowledge, sucking and swallowing and the suck:swallow ratio have not been studied in milk-fed calves.

As expected, body weight and the absolute amount of milk consumed increased significantly during the study period but the amounts relative to body weight did not change. The body weight of the three groups did not differ significantly at any measuring time. This is most likely related to the fact that only calves of group A consumed all of the milk offered (Table 1). Similarly, the calves of group C consumed an amount of milk that corresponded to only 7.1 to 8.5% of body weight per meal. The most likely reason for this was that the milk was removed when the calves stopped drinking for more than 30 sec, and thus only short-term satiation was recorded. True ad libitum feeding would in all likelihood have given different results in group C.

Large differences in the amount of milk consumed per meal by individual calves were also described in another study, in which the actual consumption ranged from 2 to 4 L when calves were offered 4 L of milk from a bottle [3]. Assuming milk consumption at the morning and evening feedings was similar, the calves of groups A, B and C drank mean daily amounts of 8.6, 9.2 and 11.0 L, respectively, in week 5, which was comparable to a daily consumption of 9 to 11 L reported for calves fed ad libitum [11, 24]. A study of 1052 preweaned calves, which were automatically fed group-housed and had a total of 40,377 healthy calf days reported a mean daily milk consumption of 6.7 L [4]. In the present study, the mean drinking duration decreased significantly from 6.4 min in week 1 to 5.2 min in week 5 and did not differ between groups. Not surprisingly, the duration of drinking was shorter when calves were fed from a bottle with a large nipple opening [3]. In contrast to the duration of drinking, the drinking speed increased significantly in the present study from 0.6 to 1.0 L/min, which was comparable to 0.9 L/min reported previously in 1052 calves [4]. A drinking speed of 1 L/min is considered ideal [25], and this may also prevent spillage of milk into the reticulorumen [26, 27]. Interestingly, the flow rate when the calf sucked a rubber teat had no linear relationship with the teat opening because calves seem to be able to adjust and control the rate of sucking when the flow rate exceeds a certain level [25]; this may also aid in prevention of leakage of milk into the reticulorumen. The drinking speed is a reliable variable for the identification of sick calves, particularly those with diarrhoea. In calves with a total of 3230 sick days, drinking speed was 0.65 L/min in contrast to 0.9 L/min in healthy calves [4].

The number of sucks/min did not change significantly during the study period. In contrast, the amount of milk ingested per suck increased an average of 35.7% from 5.6 to 7.6 ml (Fig. 1D), whereas the number of sucks/L of ingested milk decreased by 30.9% from 204 to 141 (Fig. 1C). These two variables were closely and negatively correlated at all measuring times with correlation coefficients ranging from − 0.89 to − 0.96 (Fig. 3A). The number of sucks/L of milk decreased as the volume/suck increased. Surprisingly, the number of sucks/min and the volume of a suck were significantly and negatively correlated only in week 1 (Fig. 2A).

The drinking speed was positively affected by the amount of milk ingested in one suck and negatively affected by the number of sucks/L of milk; drinking speed increased as the volume per suck increased (Fig. 4C). Conversely, drinking speed decreased as the number of sucks/L increased (Fig. 3B). We are not aware of similar findings in the literature. Drinking speed is defined as the quotient of the amount of milk consumed and drinking duration and thus is positively correlated with the former and negatively correlated with the latter.

To achieve high growth rates and maintain health, calves should be allowed to consume large amounts of milk without causing ruminal drinker syndrome. This goal is best achieved when the amount of milk ingested per suck is large. A small suck volume or a large number of sucks/L of milk is negatively correlated with the amount of milk consumed. Calves that require a large number of sucks to consume a certain amount of milk may tire faster and therefore drink less.

Of all the variables, the largest correlation coefficients were calculated for the body weights at the five measuring times. The coefficients for the correlations between the body weight in week 1 and the other weeks ranged from 0.89 to 0.96; calves with a low birth weight still had a comparatively low weight in week 5. Likewise, the numbers of sucks/L were also significantly correlated among the measuring times albeit with smaller coefficients that ranged from 0.48 to 0.70. The amounts consumed per meal in week 1 correlated with the amounts consumed in weeks 2 to 4. Lesser correlations occurred among measurements of drinking duration and drinking speed and the number of sucks/min. This suggests that these variables are affected by other factors to a greater degree than the number of sucks/min.

A limitation of this study was that the calves of group C (ad libitum) and to a lesser extent the calves of group B (16%) did not consume all of the milk offered within the specified time frame (milk was removed after the first 30-sec drinking pause). While calves of group A drank the entire amount (12.0% of body weight per day), calves of group B drank between 12.6 and 15.8% and calves of group C between 14.4 and 16.6% of body weight per day. The daily amounts consumed by calves of groups B and C did not differ in any week, which explains the small or non-existent differences between some variables in these calves.

## Conclusions

This study revealed little difference between various drinking variables in the three groups of preweaned calves offered different amounts of milk. The amount of milk consumed per meal, the drinking speed and the amount of milk ingested per suck increased significantly in all groups from week 1 to week 5, and the number of sucks/L of ingested milk decreased significantly during the same period. Numerous significant correlations between different drinking variables were determined. The amount of milk ingested per suck and the amount of milk consumed per meal were significantly correlated in the last 2 weeks but the correlation coefficients were relatively small. Weight gain was a function of drinking behaviour and other factors.

## Supplementary Information

**Additional file 1: Video 1.** A Holstein Friesian calf being fed from a bucket with a nipple. Each suck causes a clicking noise from the check valve of the nipple and the sucks are counted with a handheld tally counter.

## Data Availability

The datasets used and analysed for this study are available from the corresponding author on reasonable request.

## Abbreviations

L: Litre
Min: Minute
Fig: Figure
Kg: Kilogram
